# Spatial profiles of the bacterial microbiota throughout the gastrointestinal tract of dairy goats

**DOI:** 10.1007/s00253-024-13200-8

**Published:** 2024-06-01

**Authors:** Qingyong Hu, Jun Luo, Fei Cheng, Ping Wang, Ping Gong, Xuefeng Lv, Xinpei Wang, Min Yang, Pengbo Wei

**Affiliations:** 1 Shaanxi Provincial Key Laboratory of Agricultural Molecular Biology, College of Animal Science and Technology, Northwest A & F University, Yangling, 712100 People’s Republic of China; 2 Institute of Animal Husbandry Quality Standards, Xinjiang Academy of Animal Husbandry Science, Urumqi Xinjiang, 830000 People’s Republic of China

**Keywords:** Dairy goats, Microbial profiles, GIT, 16S rRNA, Diversity

## Abstract

**Abstract:**

The gastrointestinal tract (GIT) is stationed by a dynamic and complex microbial community with functions in digestion, metabolism, immunomodulation, and reproduction. However, there is relatively little research on the composition and function of microorganisms in different GIT segments in dairy goats. Herein, 80 chyme samples were taken from ten GIT sites of eight Xinong Saanen dairy goats and then analyzed and identified the microbial composition via 16S rRNA V1-V9 amplicon sequencing. A total of 6669 different operational taxonomic units (OTUs) were clustered, and 187 OTUs were shared by ten GIT segments. We observed 264 species belonging to 23 different phyla scattered across ten GITs, with *Firmicutes* (52.42%) and *Bacteroidetes* (22.88%) predominating. The results revealed obvious location differences in the composition, diversity, and function of the GIT microbiota. In LEfSe analysis, *unidentified_Lachnospiraceae* and *unidentified_Succinniclassicum* were significantly enriched in the four chambers of stomach, with functions in carbohydrate fermentation to compose short-chain fatty acids. *Aeriscardovia*, *Candidatus_Saccharimonas*, and *Romboutsia* were significantly higher in the foregut, playing an important role in synthesizing enzymes, amino acids, and vitamins and immunomodulation. *Akkermansia*, *Bacteroides*, and *Alistipes* were significantly abundant in the hindgut to degrade polysaccharides and oligosaccharides, etc. From rumen to rectum, α-diversity decreased first and then increased, while β-diversity showed the opposite trend. Metabolism was the major function of the GIT microbiome predicted by PICRUSt2, but with variation in target substrates along the regions. In summary, GIT segments play a decisive role in the composition and functions of microorganisms.

**Key points:**

• *The jejunum and ileum were harsh for microorganisms to colonize due to the presence of bile acids, enzymes, faster chyme circulation, etc., exhibiting the lowest α-diversity and the highest β-diversity.*

• *Variability in microbial profiles between the three foregut segments was greater than four chambers of stomach and hindgut, with a higher abundance of Firmicutes dominating than others.*

• *Dairy goats dominated a higher abundance of Kiritimatiellaeota than cows, which was reported to be associated with fatty acid synthesis.*

**Supplementary Information:**

The online version contains supplementary material available at 10.1007/s00253-024-13200-8.

## Introduction

The mammalian gut is inhabited by massive microorganisms, also known as microbiome (Kurilshikov et al. 2017). The balance of microorganisms can affect the health, production performance, reproductive performance, and nutrient utilization efficiency of the host (Mao et al. 2015). Microbial colonization occurs earliest in the embryonic stage of the uterus (Bi et al. 2021). Gut microbes are complex and variable in composition and in dynamic equilibrium with the host (Stamilla et al. 2021), which were affected by age, segment, dietary composition, season, and production stage (Li et al. 2021, 2020a; Stamilla et al. 2021).

The microbial structure of different gastrointestinal tract (GIT) sections has been intensively studied in humans, pigs, ducks, sheep, and cattle (Liu et al. 2019; Ma et al. 2022; Mao et al. 2015; Shalon et al. 2023; Zhu et al. 2020). Extensive results have shown that different GIT segments exhibited tremendous differences in microbial composition and biological functions and were highly dynamic during the life cycle. Additionally, the colonization of gut microbiota has spatial selectivity, often resulting in the selection of homologous segments for colonization (Li et al. 2020b).

The Xinong Saanen dairy goat is a cultivated breed based on the Swiss Saanen dairy goat, which is known for high milk yield, outstanding reproductive performance, and stable genetic performance. Currently, more than half of China’s dairy goats have the lineage of Xinong Saanen dairy goats (Li et al. 2022). However, research into their microbiome has tended to focus on the rumen or feces, without systematic research into the entire GITs. To fully display their genetic potential, it is necessary to decipher the microbial variation across the GITs.

For decades, 16S rRNA gene sequencing has been the dominant technique for bacterial analysis. The bacterial 16S rRNA gene contains nine variable zones (V1 to V9), which are spaced by highly conserved sequences across different taxa (Liu et al. 2019). Currently, tremendous studies were based on the short read data (i.e., reads a few hundred bases in length) due to technology restrictions (Johnson et al. 2019), which often leads to ambiguity in taxonomic classification due to different primer selections (Kuczynski et al. 2011). Today, both PacBio (Pacific Biosciences, Menlo Park, CA, USA) and Oxford Nanopore (Oxford Nanopore Technologies, Oxford, UK) sequencing platforms can routinely generate reads exceeding 1500 bp, and high-throughput sequencing of the entire 16S rRNA gene is becoming commonplace (Sereika et al. 2022; Zhang et al. 2019). Studies have shown that entire length 16S rRNA gene sequence data may accurately classify individual organisms at higher taxonomic resolution (Liu et al. 2019).

The present study investigated the microbial structure of ten different GIT sites of Xinong Saanen dairy goats using the PacBio platform, aiming to decipher typical microorganisms and functions of different GIT segments and providing insights into nutritional regulation to optimize animal performance.

## Materials and methods

### Animal feeding and sampling

Eight 4-year-old female Xinong Saanen dairy goats (body weight 57 ± 2.7 kg) in the dry period were obtained from the breeding dairy goat farm of Northwest A&F University. The dairy goats were fed the same diet and raised according to the technical specification for feeding and management of dairy goat (NY/T 2835–2015) published by Ministry of Agriculture of the People’s Republic of China (2015). Throughout both the dry and lactation periods, the goats were fed twice daily (at 06:00 and 14:00), with a same formula consisted of 49% corn silage, 21% alfalfa hay, 16.5% corn, 7.5% soybean meal, 3.6% wheat bran, 0.9% rapeseed meal, 0.45% NaCl, 0.45% CaHPO_4_, and 0.6% premix (Supplementary file 1: Table S1). Meanwhile, the dairy goats remained free from any disease and were not administered antibiotics throughout the sampling period. After fasting overnight, eight dairy goats were slaughtered after euthanasia.

After dissecting the abdomen, the digestive tract was rapidly separated and then ligated with a string at the joint of different GIT section. The chyme samples from the four stomachs were thoroughly mixed firstly and then collected using a sterile spoon. Subsequently, the chyme samples from the six intestines were taken from the middle part of the digestive tract with a sterile spoon. All samples were swiftly placed in 5-mL freezing tubes, ensuring that 80% of the cryotubes were filled, preserved in liquid nitrogen, and later stored in a − 80 °C freezer until the next step of DNA extraction (Lin et al. 2023; Mao et al. 2015). Finally, a total of 80 samples (*n* = 8/GIT segment), comprising the rumen, reticulum, omasum, abomasum, duodenum, jejunum, ileum, cecum, colon, and rectum, were harvested.

### DNA extraction and bacterial 16S rRNA sequencing

A total of 80 samples were used for 16S rRNA sequencing. Firstly, we extracted total genomic DNA from chyme samples using the cetyltrimethylammonium bromide method (LS00066, Solarbio®, Beijing, China). Next, we inspected the DNA quality by 1.0% (w/v) agarose gel electrophoresis and analyzed the DNA concentration by NanoDrop spectrophotometer (NanoDrop 2000, Thermo Scientific™, Wilmington, Delaware, USA). The V1–V9 region of the 16S rRNA gene was amplified from genome through polymerase chain reaction (PCR) with a sequential reaction program of 2 min at 95 ℃, 35 cycles of 30 s at 95 ℃, 45 s at 60 ℃, 90 s at 72 ℃, and finally a 10-min extension at 72 ℃. PCR reactions were in 50 μL system, including 5.0 μL DNA template (5 ng), 5.0 μL 5 × StimuLate, 10.0 μL 5 × Buffer, 1.0 μL forward and 1.0 μL reverse primer, 5.0 μL dNTPs (2.5 mM), 1.0 μL Trans Fastpfu, and 22.0 μL ddH_2_O. The primer sequences were as follows: 27F (AGAGTTTGATCCTGGCTCAG) and 1492R (GNTACCTTGTTACGACTT). The PCR products of barcoded V1–V9 amplicon were detected by agarose gel electrophoresis prior to processing on a PacBio Sequel II sequencing platform (Novogene, Beijing, China).

### Bioinformatic analysis

We distinguished and classified the offline data based on the different barcode sequences added during the PCR process. Next, the raw data underwent steps of primer excision, quality filtering, denoising, splicing, and chimera removal, resulting in clean reads. With the Uparse software (Uparse v7.0.1001, http://drive5.com/uparse/), we clustered clean reads into operational taxonomic units (OTUs) with 97% identity (Edgar 2013). Representative sequences of OTUs were analyzed for species annotation by the Mothur method and the SSUrRNA database from SILVA (http://www.arb-silva.de/) (Nie et al. 2023).

We calculated the α-diversity parameters and the UniFrac distance using QIIME (1.9.1) (He et al. 2018). The hierarchical tree was constructed using the unweighted paired arithmetic mean method (UPGMA) based on UniFrac distance (Wang et al. 2017). For β-diversity, Wekemo Bioincloud (Gao et al. 2024) was used to visualize the result of non-metric multidimensional scaling (NMDS), principal component analysis (PCA), and principal coordinate analysis (PCOA) based on Bray–Curtis distance.

Differential bacteria from ten GIT sites were analyzed using linear discriminant analysis (LDA) and effect size (LEfSe) software, and the default filtering value for the LDA was 4 (Wang et al. 2017). We predicted the biological functions of the GIT bacteria with Phylogenetic Investigation of Communities by Reconstruction of unobserved states (PICRUSt2) and combined with Kyoto Encyclopedia of Genes and Genomes (KEGG) data (Langille et al. 2013).

We used Spearman’s rank correlation coefficient to calculate the co-occurrence network based on the relative abundance profile of genera (Huang et al. 2018) by Wekemo Bioincloud.

### Statistical analysis

The differences of α-diversity parameters, clean reads, average length, and OTUs were compared between different GITs using the Wilcoxon rank-sum test by R (Version 4.3.1) (He et al. 2018). KEGG pathways that were significantly different (based on Tukey’s post-hoc test, *P* < 0.05) were analyzed with STAMP version 2.1.3 (Parks et al. 2014).

## Results

### Sequencing results

**Fig. 1:**
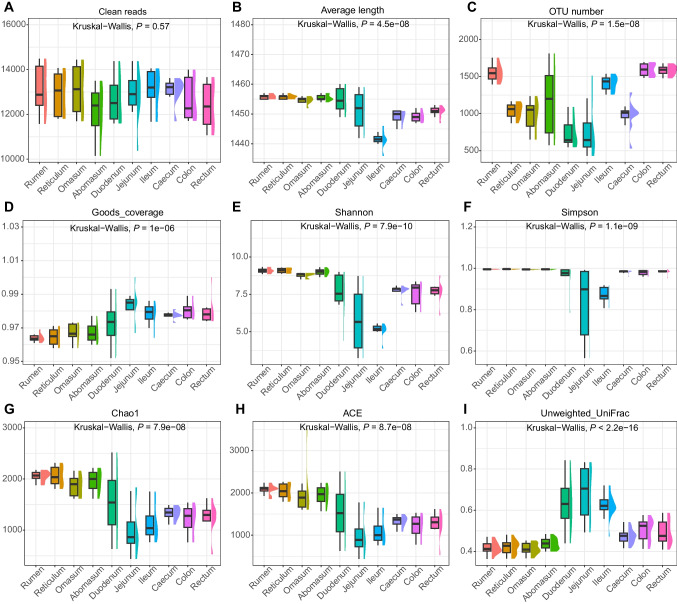
Valid sequences and diversity indexes of ten GIT microbial populations. **a** The number of clean reads sequenced from ten GIT chyme samples. **b** The average length of clean reads sequenced from ten GIT chyme samples. **c** The number of OTUs annotated to ten GIT chyme samples. **d** The goods coverage index of ten GIT chyme samples. **e** The Shannon index of ten GIT chyme samples. **f** The Simpson index of ten GIT chyme samples. **g** The Chao1 index of ten GIT chyme samples. **h** The ACE index of ten GIT chyme samples. **i** The unweighted UniFrac index of ten GIT chyme samples

A total of 1,023,714 clean reads with an average sequence length of 1452 bp were sequenced from the microbial V1–V9 region (Fig. 1A–B and Supplementary file 2 : Tables S2–S3). To investigate microbial compositional diversity, clean reads were clustered into OTUs with 97% identity, and then representative sequences of OTUs were then annotated to species.

**Fig. 2:**
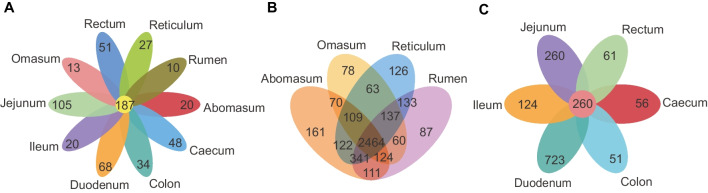
The shared OTUs of ten GIT segments. **a** The OTUs shared by ten GIT segments. **b** The OTUs shared by four chambers of stomach. **c** The OTUs shared by six intestinal segments

A total of 6669 distinct OTUs were clustered (Supplementary file 2: Tables S4–S5 and Fig. 1C), with 187 OTUs shared by ten GIT segments, and 10, 27, 13, 20, 68, 105, 20, 48, 34, and 51 OTUs were unique to the rumen, reticulum, omasum, abomasum, duodenum, jejunum, ileum, cecum, colon, and rectum, respectively (Fig. 2A). The number of OTUs that common to the four chambers of stomach was 2464, and the number of OTUs shared by the six intestinal segments was 260 (Fig. 2B–C). Segments with similar physiological functions shared more OTUs, indicating that the region had a profound effect on microbial composition.

All OTUs were classified and annotated to 23 phyla, 35 classes, 74 orders, 124 families, 264 genera and 228 species (Supplementary file 2: Tables S6–S11). There were 14 phyla, 20 classes, 28 orders, 39 families, 42 genera, and 11 species shared by ten GIT segments (Supplementary file 1: Fig. S1).

### Diversity of the GIT microbiota

We calculated the Chao1, Shannon, Simpson, ACE, Good’s coverage and unweighted UniFrac indexes to compare the diversity and richness of different GIT bacteria (Fig. 1D–I). The Good’s coverage result showed a value greater than 96.4%, implying that the sequencing depth was sufficient to assess the diversity metrics.

Among the 10 GIT segments, the 4 chambers of stomach (rumen, reticulum, omasum, and abomasum) had the highest α-diversity, followed by duodenum and the hindgut (cecum, colon, and rectum), and the jejunum and ileum had the lowest values of α-diversity (*P* < 0.05, Supplementary file 2: Tables S12–S16 and Supplementary file 1: Fig. 2A–D). The unweighted UniFrac was used to assess the β-diversity index, in contrast to the α-diversity, with the foregut having the highest β-diversity index, followed by the hindgut and finally the four chambers of stomach (*P* < 0.05, Supplementary file 2: Table S17 and Supplementary file 1: Fig. S2E).

**Fig. 3:**
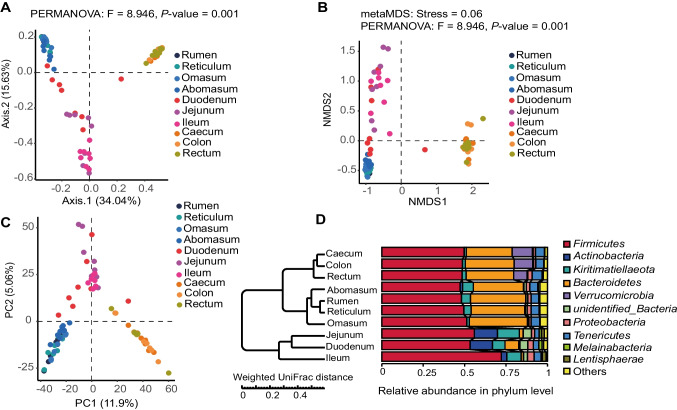
β-diversity analysis of ten GIT segments. **a** The non-metric multidimensional scaling (NMDS) plot based on Bray–Curtis distance. **b** Principal coordinate analysis (PCOA) based on Bray–Curtis distance. **c** The principal component analysis plot (PCA) based on Bray–Curtis distance. **d** The hierarchical tree shows the UPGMA clustering result

We used a PCOA plot based on the Bray–Curtis distance matrix to display the β-diversity of GIT bacteria (Fig. 3A). PCA1 explained 34.04% of the total variation, while PCA2 explained 15.63%. GIT chyme samples were divided into three groups, including the four chambers of stomach, foregut, and hindgut (*P* < 0.01). Similarly, NMDS, PCA, and UPGMA analysis demonstrated a similar trend to PCA analysis (Fig. 3B–D). These results indicated that the GIT segments with similar physiological functions have similar microbial compositions.

### Microbial profiles of ten GIT segments

**Fig. 4:**
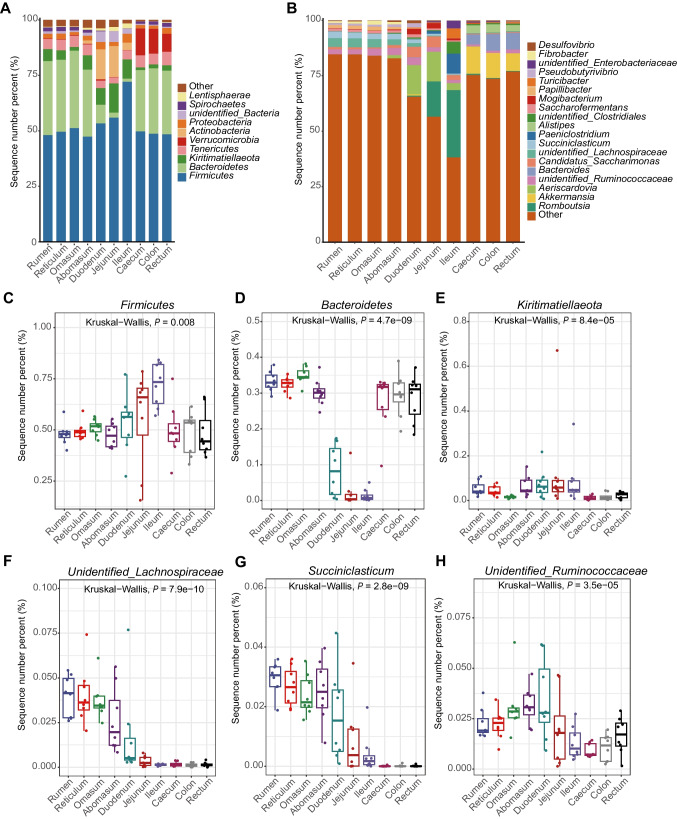
Microbial composition of the ten GIT segments. **a**The phylum-level microbial composition of each GIT segment. **b** The genus-level microbial composition of each GIT segment. **c** The *Firmicutes* composition of each GIT segment. **d** The *Bacteroidetes* composition of each GIT segment. **e** The *Kiritimatiellaeota* composition of each GIT segment. **f** The *unidentified_Lachnospiraceae* composition of each GIT segment. **g** The *Succiniclasticum* composition of each GIT segment. **h** The *unidentified_Ruminococcaceae* composition of each GIT segment

At the phylum level, the bacteria of ten GITs were predominantly classified as *Firmicutes* (52.42%) and *Bacteroidetes* (22.88%), accounting for 75.30% of the phylum classifications. There was a relative higher abundance of *Firmicutes* in the foregut than other segments. *Bacteroidetes* dominated the four chambers of stomach and hindgut, with relatively lower abundance in the foregut. In addition, *Actinobacteria* and *Kiritimatiellelaota* were abundant in the foregut, and *Verrucomicrobia* was abundant in the hindgut (Fig. 4A and C–E). In terms of genus composition, *unidentified_Lachnospiraceae*, *unidentified_Ruminococcaceae*, and *Succiniclasticum* dominated the four chambers of stomach (Fig. 4B and F–H); *Akkermansia*, *Bacteroides*, and *Alistipes* dominated the hindgut. *Aeriscardovia* and *Candidatus Saccharimonas* were prevalent in the duodenum and jejunum, whereas *Romboutsia* were abundant in the jejunum and ileum. Meanwhile, the duodenum also possessed a high abundance of *unidentified_Ruminococcaceae*, while the ileum had a high abundance of *Paeniclostridium* and *unidentified_ Clostridiales* (Fig. 4B).

These results indicated that microorganisms had a specific structure and composition within the GIT physiological region of dairy goats. Compared to the four chambers of stomach and the hindgut, the three parts of foregut had similar physiological functions, but there existed differences in the microbial structure at the genus level.

### LEfSe analysis of the GIT microbiota

**Fig. 5:**
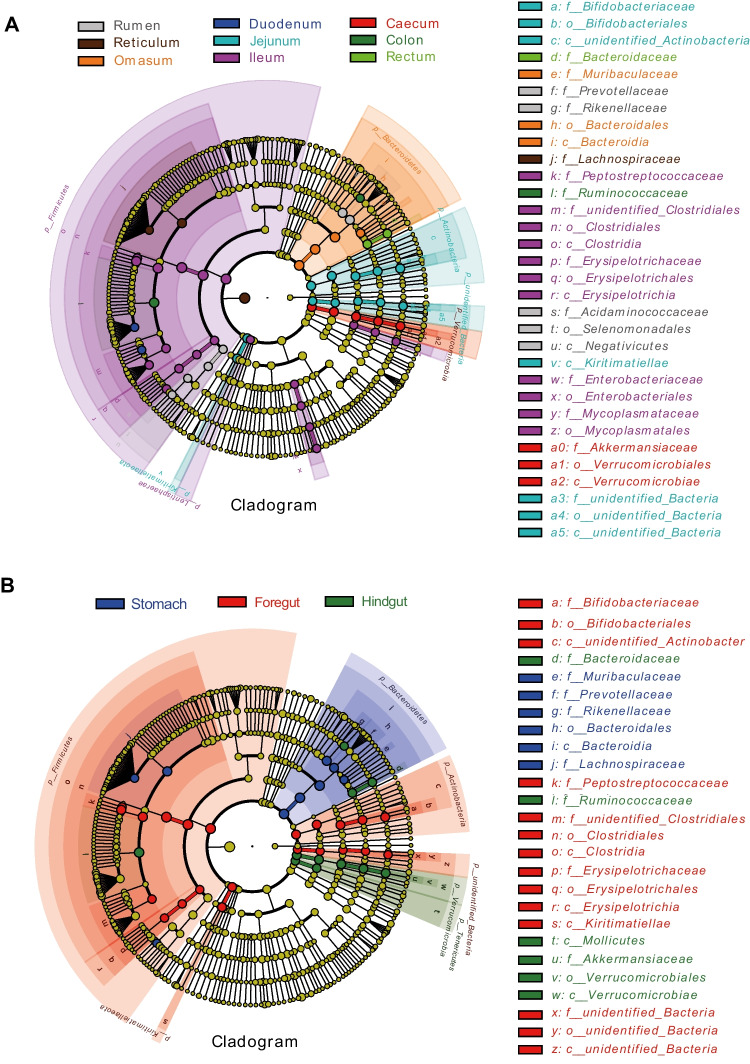
LEfSe cladogram showing the taxonomic differences of ten GIT segments. **a** LEfSe analysis of the ten GIT segment. **b** LEfSe analysis of three GIT clusters: stomach (rumen, reticulum, omasum, abomasum); foregut (duodenum, jejunum, ileum), and hindgut (cecum, colon, rectum). The node size corresponds to the average relative abundance of the taxa. Coloring principle: Taxa with no significant differences are colored in yellow, while differentiated biomarkers follow the group color

We carried out LEfSe analysis to identify differential microbiomes among ten GIT sites (LDA > 4, *P* < 0.05). We identified 56 different bacterial taxa from nine GIT fragments (Fig. 5A and Supplementary file 1: Fig. S3). LEfSe showed the dominant differential bacterial taxa of each GIT parts, which were 6 taxa in the rumen (e.g., f__*Rikenellaceae*), 3 taxa in the reticulum (e.g., f__*Lachnospiraceae*), 4 taxa in the omasum (e.g. c__*Bacteroidia*), 2 taxa in the duodenum (e.g., g__*Mogibacterium*), 12 taxa in the jejunum (e.g. p__*Actinobacteria*), 20 taxa in the ileum (e.g., f__*Peptostreptococcaceae*), 5 taxa in the caecum (e.g. o__*Verrucomicrobiales*), 2 taxa in the colon (e.g., f__*Ruminococcaceae*), and 2 taxa in the rectum (e.g. g__*Bacteroides*).

In addition to the differential taxa of each GIT segment, at the genus level, we revealed that *unidentified_Lachnospiraceae* and *Succinniclassicum* were significantly abundant in the four chambers of stomach; *Romboutsia, Aeriscardovia*, *Paeniclostridium*, and *Candidatus Saccharimonas* were significantly dominated in the foregut; and *Akkermansia, Bacteroides*, and *Alistipes* were significantly abundant in the hindgut (Fig. 5B and Supplementary file 1: Fig. S4).

### Co-occurrence network of the GIT microbiome

**Fig. 6:**
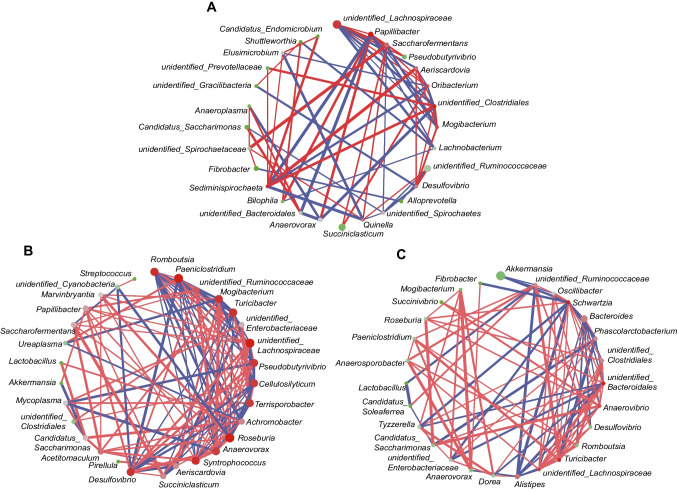
GIT microbial co-occurrence network analysis. **a** The co-occurrence of microbiota in the four chambers of stomach. **b** The co-occurrence of microbiota in the foregut. **c** The co-occurrence of microbiota in the hindgut. Red line: Spearman’s rank correlation coefficient > 0.40. Blue line: Spearman’s rank correlation coefficient <  − 0.40. The size of nodes was proportional to the relative abundance of genera; the color of nodes was the level of closeness centrality (red, high; grey, medium; green, low)

To explore the interaction between microorganisms in the GIT, we built a co-occurrence network based on the genera level (Fig. 6A–C). In the four charmers of stomach, 28 genera formed an interaction map, where *Papillibacter*, *unidentified_Clostridiales*, *Sediminispirochaeta*, and *unidentified_Lachnospiraceae* were the core genus based on the closeness centrality. Meanwhile, there was a positive correlation between *unidentified_Clostridiales* and *Sediminispirochaeta*, as well as *Papillibacter* and *unidentified_Lachnospiraceae.* However, *Papillibacter* was negatively correlated with *unidentified_Clostridiales* and *Seminispirochaeta*, and* unidentified_Clostridiales* was negatively correlated with *unidentified_Lachnospiraceae.* In the foregut, 30 genera formed an interaction map, and *Roseburia*, *Paeniclostridium*, and *unidentified_Lachnospiraceae* were the core genus. *Roseburia* and *Paeniclostridium* showed a positive correlation, and they were negatively correlated with *unidentified_Lachnospiraceae*. In the hindgut, 27 genera formed an interaction map, and *Schwartzia, unidentified_Bacteroidales*, and *Turicibacter* were the core genus, and they were positively correlated with each other.

### Predicted biological functions of the GIT microorganisms

**Fig. 7:**
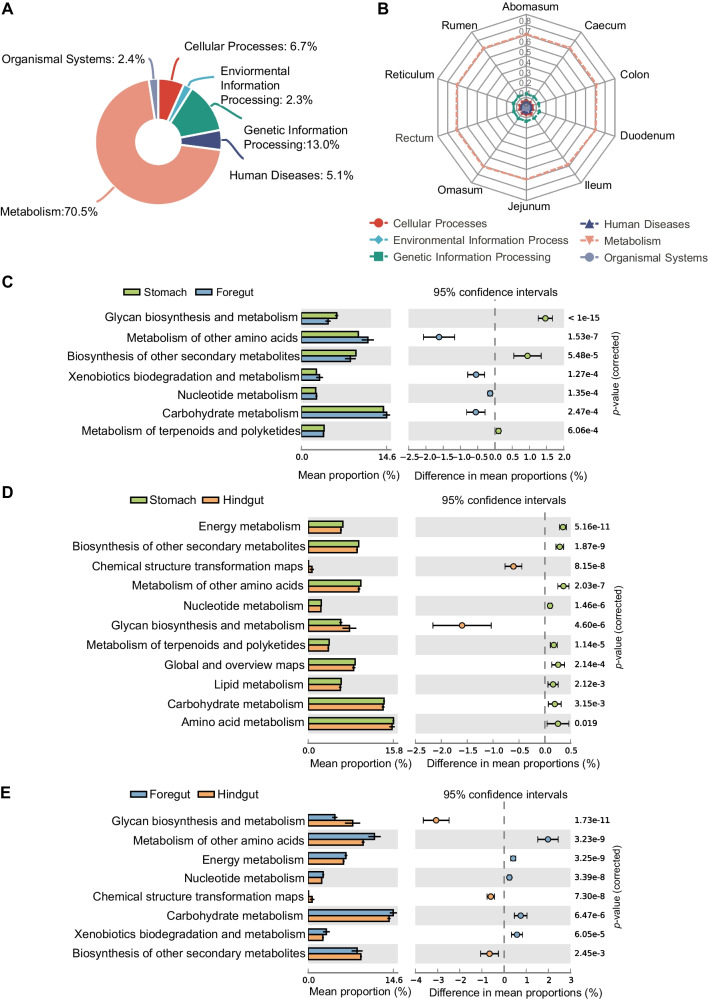
Distribution of the predicted functional pathway **a** Biological functions enriched based on KEGG level 1 of ten GITs. **b** Biological functions enriched based on KEGG level 1 in different GITs. **c** The differential metabolic pathways between stomach and foregut based on the KEGG level 2. **d** The differential metabolic pathways between stomach and hindgut based on the KEGG level 2. **e** The differential metabolic pathways between foregut and hindgut based on the KEGG level 2. Stomach (rumen, reticulum, omasum, abomasum); foregut (duodenum, jejunum, ileum), hindgut (cecum, colon, rectum)

We utilized PICRUSt2 to predict the functional profiles of bacteria across different segments of the GIT, and our findings indicated that metabolism was the predominant enriched functions. At KEGG level 1, six major pathways were represented across GIT regions, including metabolism (70.5%), genetic information processing (13.0%), and environmental information processing (2.3%) (Supplementary file 2: Table S18 and Fig. 7A–B).

At KEGG level 2, the top five identified functions were related to amino acid metabolism (11%), metabolism of cofactors and vitamins (10%), carbohydrate metabolism (10%), metabolism of other amino acids (7%), and biosynthesis of other secondary metabolites (6%) (Supplementary file 2: Table S19). Compared to the foregut, the functional abundance of the four chambers of stomach and the hindgut was abundant in glycan biosynthesis and metabolism and biosynthesis of other secondary metabolites. Compared to the hindgut, the functional abundance of the four chambers of stomach was prevalent in energy metabolism, amino acid metabolism, and carbohydrate metabolism but lower in glycan biosynthesis and metabolism. Compared with the hindgut, the functional abundance of the foregut was advantageous in the metabolism of other amino acids, carbohydrate metabolism, and energy metabolism (Fig. 7C–E).

In addition, it was worth noting that at the level of KEGG level 3, in terms of carbohydrate metabolism, the advantage of the foregut laid in starch and sucrose metabolism, amino sugar, and nucleotide sugar metabolism. In terms of glycan metabolism, the advantage of the hindgut laid in glycosaminoglycan degradation and other glycan degradation. In terms of lipid metabolism, the foregut had an advantage in glycerolipid metabolism and glycerophospholipid metabolism; the hindgut was abundant in steroid biosynthesis; the four chambers of stomach were mainly involved in fatty acid biosynthesis and linoleic acid metabolism (Supplementary file 2: Table S20).

## Discussion

In recent decades, the GIT microbial community has been identified as an essential “superorganism,” playing fundamental roles in immunity, digestion, metabolism, and brain–gut communication, which were essential to maintain balanced gut physiology and host health (Aziz et al. 2013). Herein, we performed third-generation 16S rRNA full-length sequencing to investigate the composition and function of ten GIT segments. To our knowledge, this was the first time that third-generation sequencing technology has been used to explore the microbial composition of the whole GIT sections in dairy goats.

First, a total of 1,023,714 clean reads with an average length of 1452 bp were sequenced from the microbial V1–V9 region, and 6669 distinct OTUs were ultimately identified, which were higher than previously sequenced on sheep (322–789 OTUs) (Wang et al. 2017) and goats (1118 OTUs) (Li et al. 2022), indicating that we were able to investigate the bacterial community with a higher resolution.

GIT is a multi-segmented biological system in anatomy, where oxygen concentration, available nutrients, pH value, bile acids, chyme circulation time, mucus, and immunity are important determinants of microbial colonization (Friedman et al. 2018). The present study elucidated that the richness and diversity index of the GIT microbiome went down first from the four chambers of stomach to the foregut and then went up in the hindgut, which was consistent with previous studies in dairy cattle (Lin et al. 2023). Studies have confirmed that the microbial diversity and richness of foregut were significantly lower than hindgut of Aohan fine wool sheep (Ma et al. 2022). In the foregut, with a faster transit time, the presence of antimicrobial compounds, such as bile acids and digestive enzymes, makes it difficult for microorganisms to colonize and establish a stable flora. However, the flow rate of chyme in the hindgut is slower, and the metabolism is conducive to fermenting undigested plant materials or polysaccharides in host mucus, resulting in a more abundant species (Martinez-Guryn et al. 2019; Tropini et al. 2017).

The results of the PCOA, NMDS, PCA, and UPGMA analysis revealed that ten GIT segments were clustered into three groups, including the four chambers of stomach, the hindgut and the foregut, which was consistent with the previously reported results (Lin et al. 2023; Ma et al. 2022). However, it reported that the microbial structure of the duodenum of the Qinghai semi-fine wool sheep was separate from the other intestinal segments (Wang et al. 2021). This may be related to the unique physiological environment of the duodenum (Martinez-Guryn et al. 2019) and the unique location of the duodenum, which connected the stomach and distal intestinal segments (Wang et al. 2021).

Different regions of the GIT house extensive gut microbes and provide diverse functions (Martinez-Guryn et al. 2019). High proportions of *Firmicutes* and *Bacteroidetes* were found in all GIT segments, consistent with findings in dairy cattle (Lin et al. 2023) and sheep (Wang et al. 2021), but different from those in horses (Costa et al. 2015) and camels (He et al. 2018). Although *Firmicutes* was the first dominant genus, the second dominant genus varied depending on species, with camels being *Verrucomicronia* and horses being *Verrucomicronia* or *Proteobacterium* depending on the GIT region. Previous studies have demonstrated that *Firmicutes* could metabolize dietary plant polysaccharides (David et al. 2014), and members of the *Bacteroidetes* predominately colonized in the distal intestine, encoding a large number of carbohydrate-degrading active enzymes (CAZYmes such as glycoside hydrolases and polysaccharide lyases), which were able to utilize dietary both dietary and host mucosal glycans (El Kaoutari et al. 2013; Johnson et al. 2017).

Moreover, our research has shown that the foregut had a higher percentage of *Firmicutes* and a lower proportion of *Bacteroidetes*. Bile acids secreted by the pancreas converge in the foregut. Studies have shown that the relative abundance of *Firmicutes* in the rat cecum was increased, whereas the relative abundance of *Bacteroidetes* was decreased due to dietary cholic acids (Islam et al. 2011), which was consistent with our findings. In addition, *Actinobacteria* were dominant in the foregut, where they play a key role in regulating intestinal barrier function, secreting fiber-digesting enzymes, maintaining the body’s immunity and participating in the gut-brain axis communication (Binda et al. 2018).

Strikingly, *Kiritimatiellaeota* ranked third in phylum composition, with an average abundance of 5.24% (Supplementary file 2: Table S3) and was associated with high milk fat content and short-chain fatty acids production (Guo et al. 2022; Stergiadis et al. 2020). Compared to dairy cows, there were few reports of *Kiritimatiellaeota* (Amin et al. 2023; Lin et al. 2023; Mao et al. 2015), which may be related to the unique physiological characteristics of dairy goats, with higher goat milk fat than cow’s milk (Collard and McCormick 2021). *Kiritimatiellaeota* serves as a characteristic phylum of dairy goats to produce potential substrates for synthesizing milk fat, but further validation is needed (Supplementary file 1: Fig. S5).

The hindgut showed a greater percentage of *Verrucomicrobia* than other segments. *Verrucomicrobia* can decompose polysaccharides to provide energy and nutrients (Sichert et al. 2020) and compose short-chain fatty acids (such as propionic acid and butyric acid), which play an essential role in regulating GIT health and the immune system (Lindenberg et al. 2019).

In addition, this study demonstrated that 41 genera were shared by ten GIT segments. However, a high mass of *unidentified_Lachnospiraceae*, *unidentified_Ruminococcaceae*, and *Succiniclasticum* was observed in the four chambers of stomach. Earlier studies have reported that the first most abundant genus was *Prevotella* in the four chambers of stomach in dairy cattle, sheep, and camels, and the second most abundant genera was *Fibrobacter* in dairy cattle and camel and unidentified *Ruminococcaceae* in sheep (He et al. 2018; Li et al. 2022; Lin et al. 2023).

In our study, the proportion of *Prevotella* was relatively low, which could be related to the animal status, diet composition, and sequencing methods. Firstly, it has been reported that the *Prevotella* genus increased from the dry milk stage to the lactation stage (Zhu et al. 2017). Similarly, our experimental animals were in the dry milk stage. Secondly, the *Prevotella* family accounted for 11.28% of the total in the rumen; however, the *Prevotella* genus was 0.28%, which may be related to the 16S rRNA gene full-length sequencing technology, which identified more other genera and reduced their relative proportion; finally, research has reported that diet composition, and feed efficiency may affect *Prevotella* abundance (Ellison et al. 2017).

*Lachnospiraceae* was considered beneficial bacteria in the human gut because it could ferment carbohydrates into short-chain fatty acids (Duncan et al. 2007). *Ruminococcaceae* were thought as the manufacturer of SCFAs (Xie et al. 2022) and were involved in fat degradation and fatty acid β-oxidation (Baars et al. 2018; Zhang et al. 2023). *Succiniclasticum* was a propionate producer and showed lower abundance in high efficiency animals (Manzanares-Miranda et al. 2023).

*Aeriscardovia*, *Candidatus_Saccharimonas*, and *Romboutsia* tended to be more abundant in the foregut, which were consistent with the reported high proportion of *Candidatus Saccharimonas* in the ileum and jejunum of sheep (Wang et al. 2021). However, the dominant genera in the foregut reported in pigs were *Lactobacillus* and *Escherichia* (Liu et al. 2019) and *Phyllobacterium* and *Eubacterium* in dairy cattle (Lin et al. 2023), indicating that species have a crucial influence on their GIT microbial composition. Interestingly, studies in pigs have shown that the microbial composition of the duodenum and jejunum was similar, while the ileum was a separate group, which was consistent with our study, suggesting that GIT regions have a pivotal impact on microbial colonization.

*Aeriscardovia* was capable of self-synthesizing a wide variety of enzymes, amino acids, and vitamins by itself and metabolize substances, such as acetate and lactic acid (Zhu et al. 2022). Studies showed the relationship between *Romboutsia* and reduced plasma pro-inflammatory cytokine levels (Li et al. 2018). *Candidatus_Saccharimonas* was effective in the immune recovery of immune deficient patients (Xie et al. 2021). All these findings pointed to a contribution of the foregut microbiota to host immunity.

*Akkermansia*, *Alistipes*, and *Bacteroides* showed a higher abundance in the hindgut. This was consistent with reports in dairy cattle where *Bacteroides* and *Alistipes* were prevalent in the hindgut (Lin et al. 2023), and consistent with a report in camels where *Akkermansia* was enriched in the hindgut (He et al. 2018), but differed from the reported high proportion of *Ruminococcaceae UCG-005* in the hindgut of sheep (Wang et al. 2021). *Akkermansia* spp*.* isolated from the mammalian GIT chyme and fecal contents had functions in synthesizing enzymes to deconstruct and utilize mucin in the GIT (Gong et al. 2020; Tailford et al. 2015) and showed an anti-obesity protective effect via modulation of glucose metabolism (Zhou et al. 2020). *Alistipes* was reported to utilize host-derived glycans and was dominant in the hindgut of dairy cattle (Lin et al. 2023). *Bacteroides*, a phylum of bacteria that metabolize polysaccharides and oligosaccharides, could supply nutrients and vitamins to its host and other GIT microbial microorganisms (Zafar and Saier 2021). Thus, the ecological location of the GIT is a decisive funnel that selects microbial populations through nutrients, oxygen concentration, and host metabolites, etc. (Tropini et al. 2017).

In conclusion, the microbiome of four chambers of stomach evolved towards decomposing plant-derived glycans, and the post-gastric microbiome towards to utilize host-derived glycans, which was consistent with the findings in dairy cows (Lin et al. 2023).

Microbial co-occurrence network showed complicated interactions between core genera, where they competed or collaborated with each other. And the core bacterial genera varied along GITs, which was related to the physiological characteristics and the available nutrients. Contrary to the reports on chicken, the microbial interactions in the foregut are not as complex as those in the hindgut, and *Lactobacillus* was the major genera and negatively correlated with other genera (Huang et al. 2018). This study demonstrated a complicated microbial network of foregut and other segments, which was consistent with the report on chicken (Wen et al. 2021) and cow (Ji et al. 2018).

Based on the prediction of PICRUSt2, metabolism was the main biological function enriched, which was consistent with previous studies (Ma et al. 2022; Wang et al. 2021). The hindgut and four chambers of stomach underwent more glycan biosynthesis and metabolism than the foregut, which may be related to the presence of *Bacteroidetes, unidentified_Lachnospiraceae*, *unidentified_Succiniclasticum*, and *Alistipes*. In contrast, the foregut showed higher functions of metabolism of other amino acids, which was associated with a higher abundance of *Aeriscardovia.* These findings revealed that segmental differences in microbial functional groups in the GIT, which may be related to community composition and microbiome interactions in different GIT segments (Chevrette et al. 2022).

Collectively, the current study deciphered the bacterial composition and potential biological functions at different GIT locations in the Xinong Saanen dairy goats based on 16S rRNA gene full length sequencing. Furthermore, we observed significant differences in microbial diversity and relative taxa abundance among the ten GIT segments. The GIT chyme samples were classified into three different groups corresponding to relative physiological regions by β-diversity analysis. PICRUSt2 revealed compartmentalized differences in GIT microbial function. Taken together, these results elucidate the profiles of the GIT microbiota and facilitate to optimize the health and milk production of dairy goats.

## Supplementary Information

Below is the link to the electronic supplementary material.

Supplementary file1 (PDF 710 KB)

Supplementary file2 (XLSX 1001 KB)

## Data Availability

The authors confirm that the data supporting the findings of this study are available within the supplementary materials. Raw sequence reads for all samples are available under NCBI Sequence Read Archive database with accession number PRJNA1065510.
