# Supplementary material for: Spatial profiles of the bacterial microbiota throughout the gastrointestinal tract of dairy goats
Source: Appl Microbiol Biotechnol. 2024 Jun 1;108:356. doi: 10.1007/s00253-024-13200-8 (PMC11144141; doi:10.1007/s00253-024-13200-8)
Supplement: Supplementary file 1 [file 253_2024_13200_MOESM1_ESM.pdf]

Supplemental Material

Journal name

Applied Microbiology and Biotechnology

Manuscript Title

Spatial profiles of the bacterial microbiota throughout the gastrointestinal tract of dairy goats

The name(s) of the author(s):

Qingyong Hu [1], Jun Luo [1*], Fei Cheng [1], Ping Wang [1], Ping Gong [2], Xuefeng Lv [2], Xinpei Wang [1], Min Yang [1], Pengbo Wei [1]

The affiliation(s) and address(es) of the author(s):

[1]Shaanxi Provincial Key Laboratory of Agricultural Molecular Biology, College of Animal Science and Technology, Northwest A & F University, Yangling 712100, PR China

[2]Institute of Animal Husbandry Quality Standards，Xinjiang Academy of Animal Husbandry Science，Urumqi Xinjiang 830000, PR China

The e-mail address, telephone and fax numbers of the corresponding author

* Corresponding author: Jun Luo. E-mail: luojun@nwafu.edu.cn

Table S1 Chemical composition and nutrient levels of the diet (air-dry basis)

Items	Percent (%)
Ingredient	
Corn	15.40
Wheat Bran	4.80
Soybean meal	12.00
Rapeseed meal	5.00
Salt	0.80
Dicalcium phosphate	0.80
Premix (1)	1.20
Corn silage	32.86
Alfalfa hay	27.14
Nutrient levels (2)	
Metabolic energy（MJ/Kg）	8.74
Crude protein	15.31
Ether extract	2.32
Neutral detergent fiber	39.93
Acid detergent fiber	25.77
Calcium	0.53
Phosphorus	0.35

(1) The premix provides the following per kg of diets: VA 100,000 IU; VD_3 40,000 IU; VE 300 IU; Mn (as manganese sulfate) 600 mg; Zn (as zinc sulfate) 1000 mg; Cu (as copper sulfate) 200 mg; Fe (as ferrous sulfate) 1100 mg.

(2) All of the data are analyzed values except for metabolic energy.

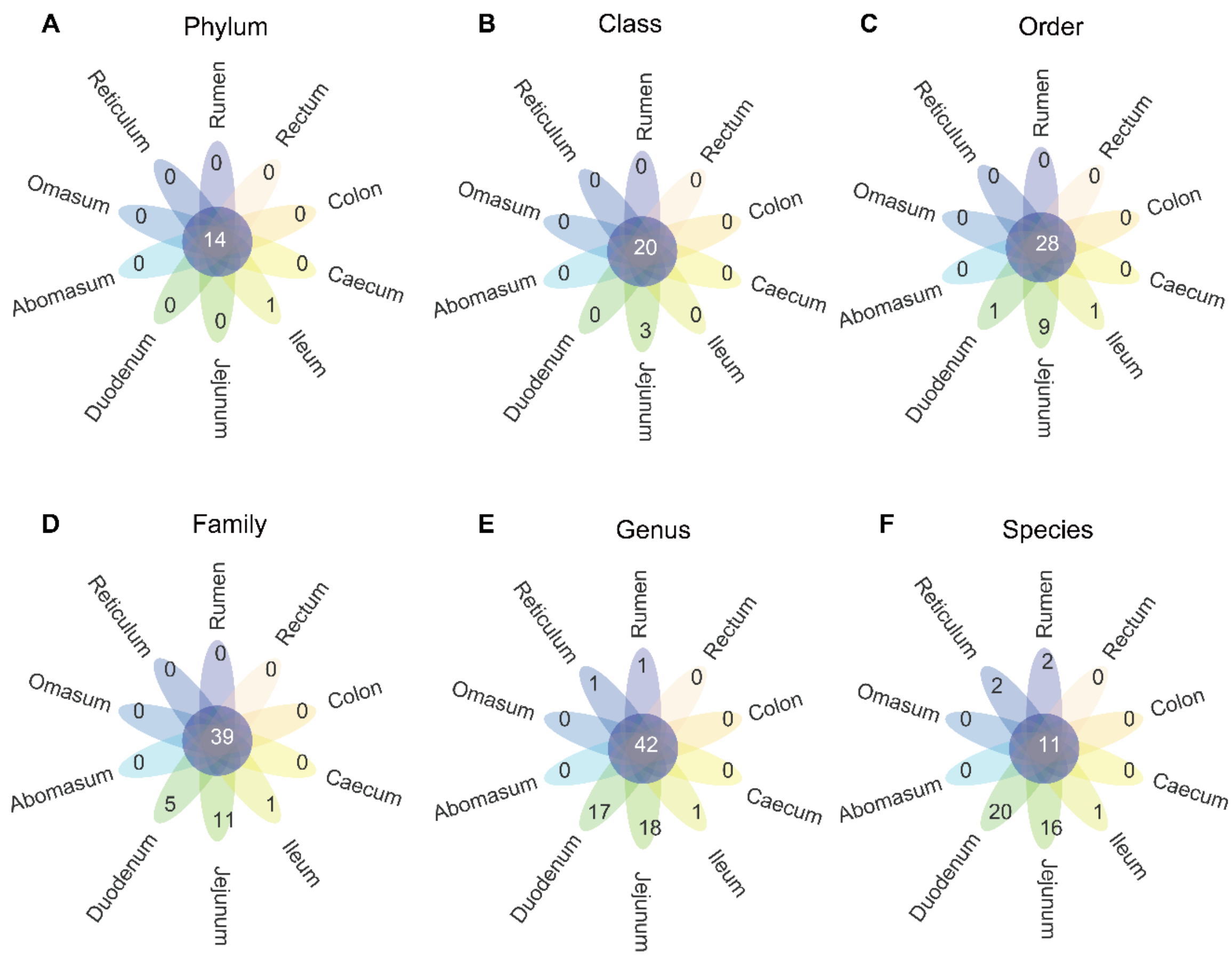

Fig. S1 The shared taxon among the GIT segments. (a) Venn diagram showing the phylum shared among ten GIT segments. (b) Venn diagram showing the class shared among ten GIT segments. (c) Venn diagram showing the order shared among ten GIT segments. (d) Venn diagram showing the family shared among ten GIT segments. (e) Venn diagram showing the genus shared among ten GIT segments. (f) Venn diagram showing the species shared among ten GIT segments.

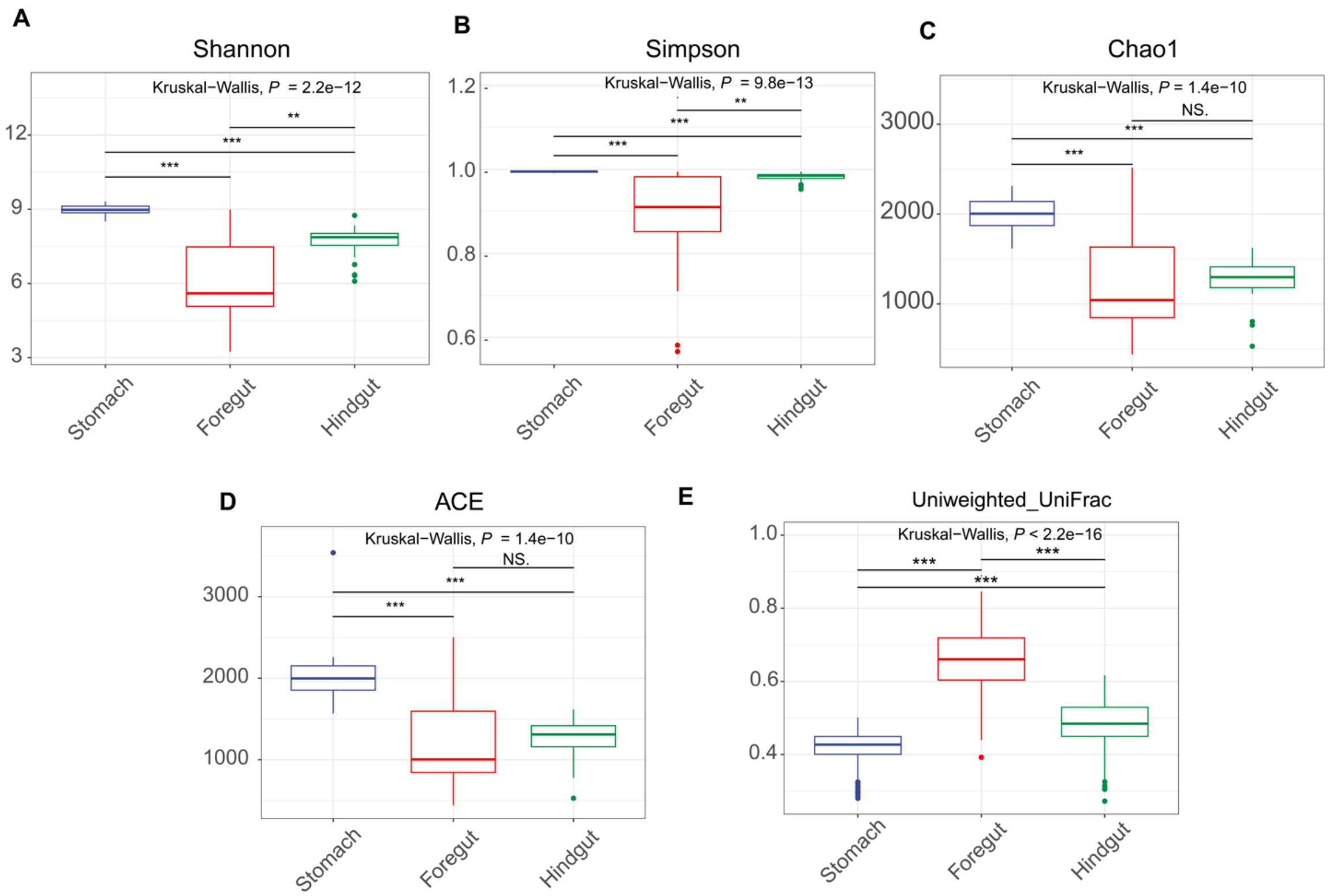

Fig. S2 α-diversity indexes and unweighted UniFrac index of three GIT clusters, Stomach (rumen, reticulum, omasum, abomasum); Foregut (duodenum, jejunum, ileum); Hindgut (cecum, colon, rectum). (a) The Shannon index of three GIT clusters. (b) The Simpson index of three GIT clusters. (c) The Chao1 index of three GIT clusters. (d) The ACE index of three GIT clusters. (e) The unweighted UniFrac index of three GIT clusters.

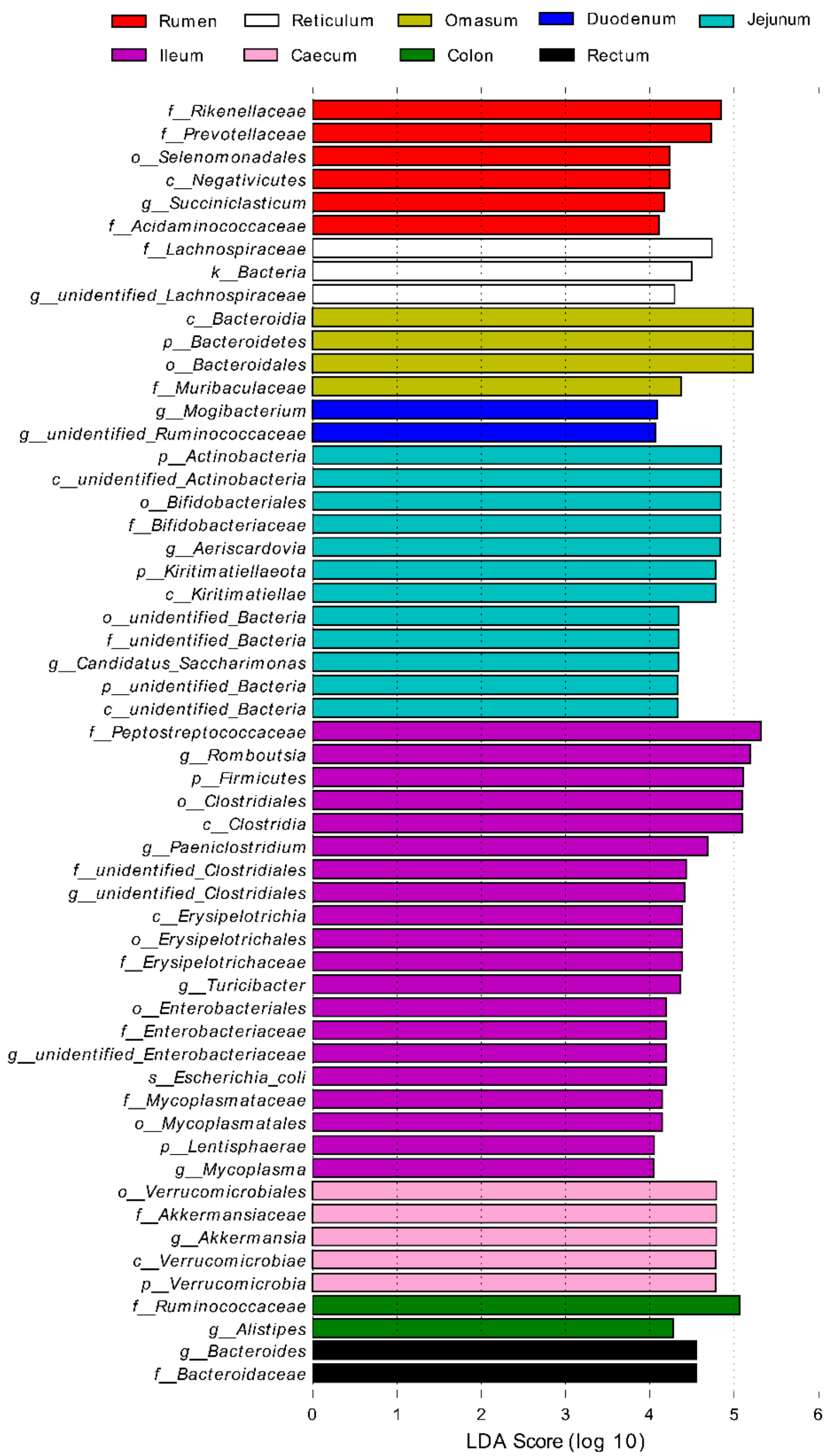

Figure. S3 The LEfSe analysis histogram of ten GIT segments. The ordinate is the taxa with significant differences between groups, and the abscissa is a bar graph displaying the LDA logarithmic score value of each taxon. The longer the length, the more significant the difference of the taxon.

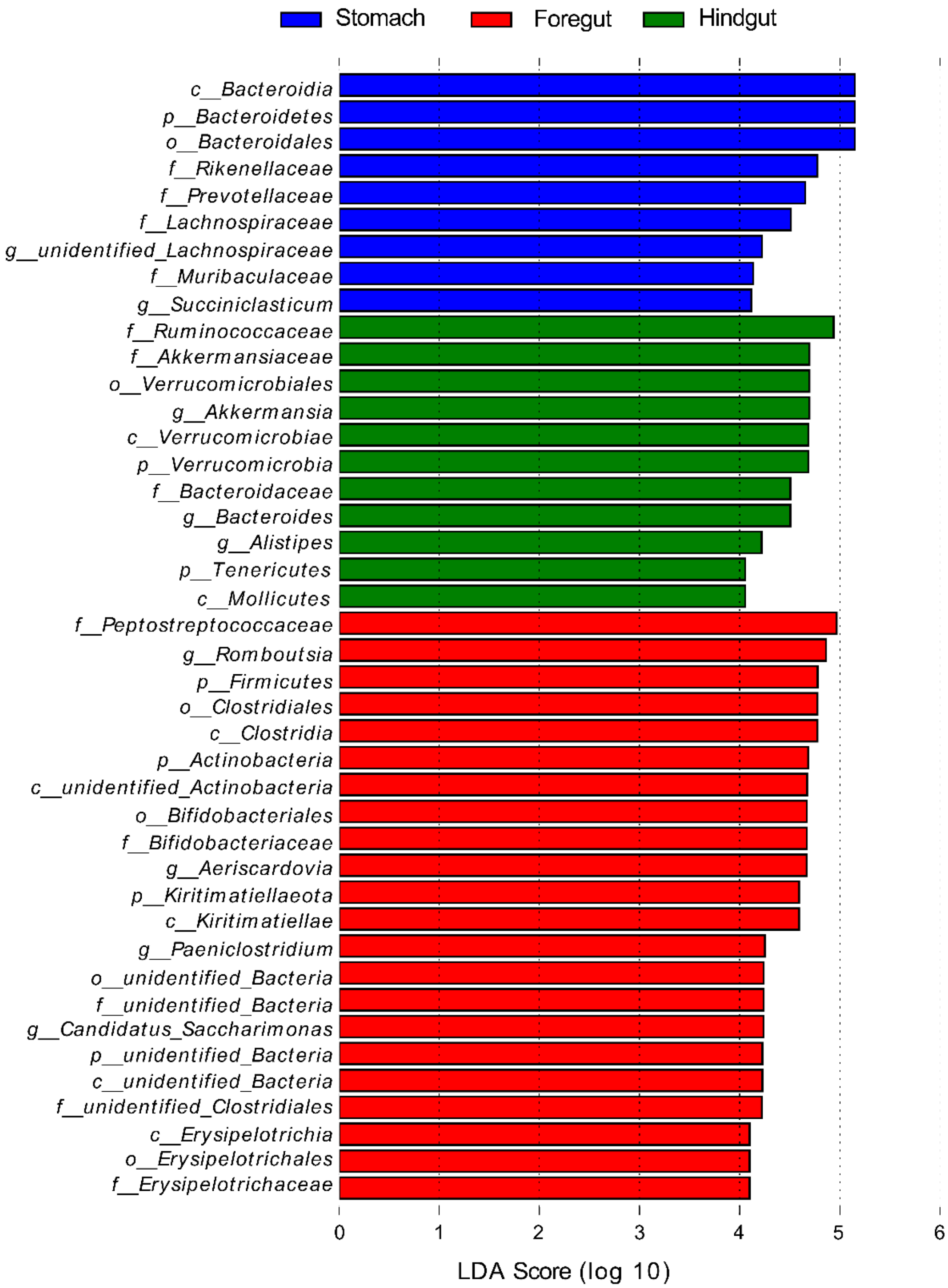

Figure. S4 The LEfSe analysis histogram of three GIT clusters, Stomach (rumen, reticulum, omasum, abomasum); Foregut (duodenum, jejunum, ileum); Hindgut (cecum, colon, rectum). The ordinate is the taxa with significant differences between groups, and the abscissa is a bar graph displaying the LDA logarithmic score value of each taxon. The longer the length, the more significant the difference of the taxon.

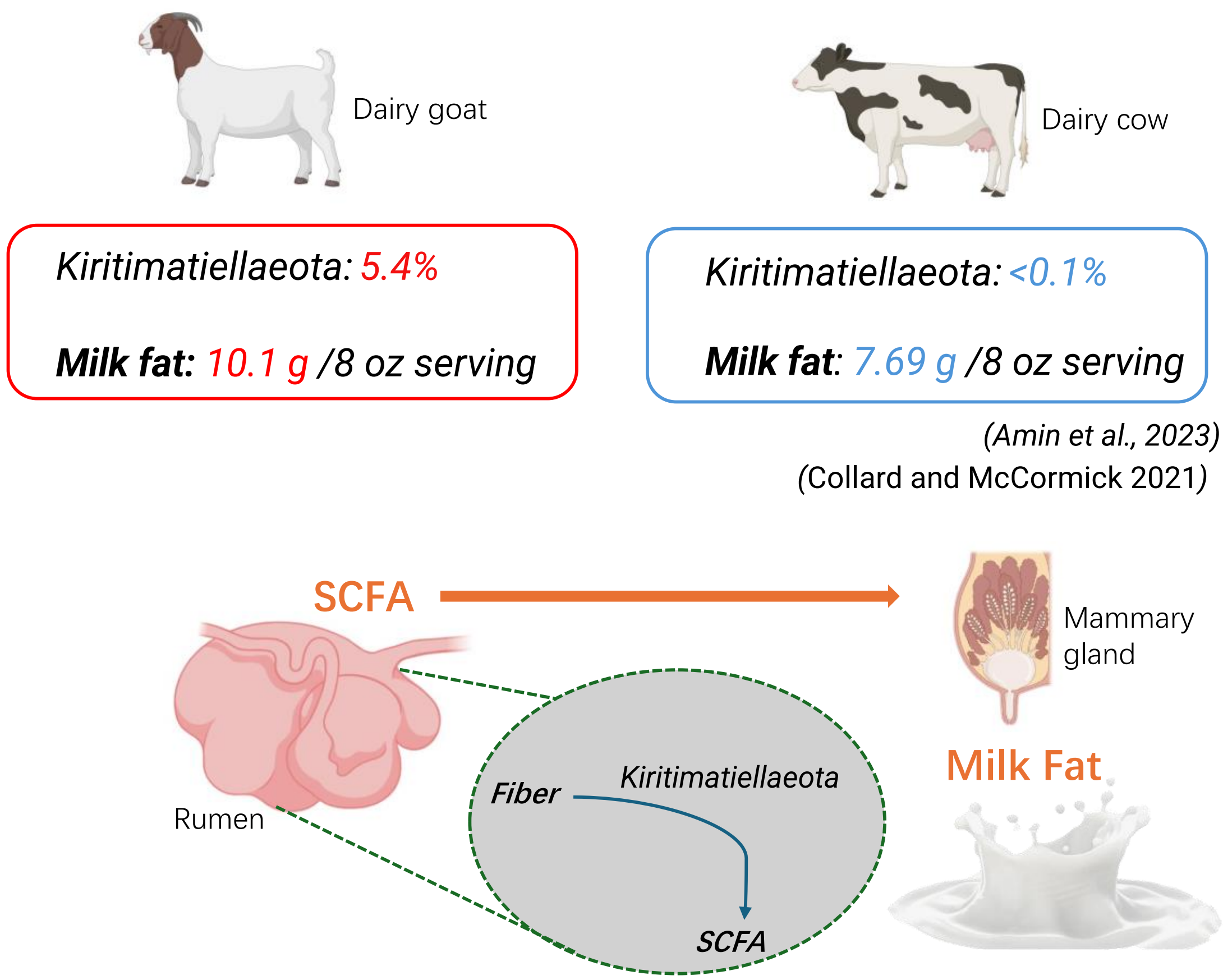

Figure. S5 Potential causes of milk fat inconsistency caused by differences in rumen *Kiritimatiellaeota* between dairy goats and dairy cows.
